# A RE-AIM Analysis of an Intergenerational Dementia Education Program

**DOI:** 10.3389/fpubh.2020.00248

**Published:** 2020-07-03

**Authors:** Ashleigh E. Smith, Georgina L. Kamm, Samantha Lai, Melissa J. Hull, Jess R. Baker, Rachel Milte, Julie Ratcliffe, Tobias Loetscher, Hannah A. D. Keage

**Affiliations:** ^1^Alliance for Research in Exercise, Nutrition and Activity (ARENA) Research Concentration, Allied Health and Human Performance, University of South Australia, Adelaide, SA, Australia; ^2^Cognitive Ageing and Impairment Neurosciences (CAIN) Research Group, Justice and Society, University of South Australia, Adelaide, SA, Australia; ^3^School of Psychiatry, Liverpool Hospital, University of New South Wales, Kensington, NSW, Australia; ^4^Health and Social Care Economics Group, College of Nursing and Health Sciences, Flinders University, Adelaide, SA, Australia

**Keywords:** intergenerational program, school-based, dementia-friendly communities, program evaluation, RE-AIM

## Abstract

**Objectives:** Children often have a lack of dementia understanding and poor attitudes toward people with dementia. Intergenerational programs are increasingly common, but the effects on knowledge and attitudes related to dementia are mixed, especially in the long-term (6 months). Using a RE-AIM framework, we quantitatively evaluated the effects of an educational dementia program (with and without an intergenerational program) on dementia attitudes in the short and long-term, and qualitatively, which elements of the program facilitated this change.

**Methods:** Eighty-one children (9.63 ± 0.52 years, 35 males) from three classes participated in an 8-week dementia education program and 52 also interacted with older adults through an intergenerational experience. Program reach was measured as the percentage of children who participated in the study. The Kids Insight into Dementia Survey (KIDS) was implemented to measure dementia knowledge and attitudes: efficacy and maintenance. Qualitative interviews with all participant groups informed both adoption and implementation. Cost-benefit analysis was used as a secondary outcome measure for efficacy.

**Results:** The program demonstrated strong levels of impact reaching 93% of school children across the three included classes. Efficacy was demonstrated by a positive change in children's dementia knowledge and attitudes immediately post program, which remained increased (as compared to baseline) 6- months post intervention; there were no differences between groups (those who interacted with older adults and those who did not). Interviews identified positive changes in children's empathy and improved community awareness. Barriers to adoption included the project scope, time constraints incurred by school terms and the management of children-to-adult ratios.

**Conclusions:** These findings provide the first evidence that school-based dementia education improves knowledge of and attitudes toward people with dementia long-term. We demonstrated programs such as this can be successful in both primary school and wider community settings, with support from school and community partners key to the success.

## Introduction

People living with dementia often experience loneliness and stigma, potentially leading to a withdrawal from social activities and delays in seeking a formal diagnosis (1). A lack of community-level dementia knowledge can contribute to misinformation about dementia and its risk factors, as well as propagate unhelpful attitudes and stigmatized views. Evidence suggests that the general public have varying levels of dementia knowledge (2, 3). This knowledge may be influenced by gender, education, or current caring responsibilities of an older adult or a person living with dementia (4, 5). With an increase in the number of people living with dementia worldwide, it is essential that the community have adequate dementia knowledge.

Community education programs have been developed and implemented to improve dementia knowledge and attitudes. Target populations for these programs have typically been general practitioners (6), public health and service workers (7), aged care staff and family members of people with dementia (8). One segment of the population that may be particularly receptive to information about dementia, yet are understudied, are children; who are at an age where their health beliefs and attitudes are malleable (9).

According to one government survey, as many as one-third of British children aged 8–17 years knew someone living with dementia, but two-thirds indicated a lack of dementia knowledge prevented them from assisting these individuals (10). Similarly, in a recent qualitative study, children's misconceptions about dementia were evident in the words they used to describe someone living with dementia, such as “frustrating” and “crazy” (11). To address this lack of knowledge, school-based dementia education programs are now being introduced with success (12, 13). These programs have successfully increased children's dementia knowledge and attitudes at their conclusion compared to the start. Whether this knowledge and attitudinal shift is retained in long-term (e.g., over months), remains unknown.

A second evidence-based method used to increase children's dementia knowledge and attitudes is engaging children and older adults together in structured intergenerational programs (14). The programs are typically mutually beneficial for all participants. Increased activity engagement and reduced social isolation, depression and anxiety has been reported for older participants (15, 16). While the younger participants report more positive attitudes toward people living with dementia, improved social skills, and increased self-confidence (17, 18). Arguably, combining both school-based dementia education alongside an intergenerational program may augment any independent increases in children's dementia knowledge and attitudes. However, to our knowledge, no study has investigated if the addition of an intergenerational component delivered alongside dementia education leads to cumulative benefits on dementia knowledge and attitudes long-term, in school-aged children.

The purpose of this study was to evaluate an 8-week school-based dementia education program with 6-weeks of embedded intergenerational interaction using the RE-AIM framework (19). The program was co-designed and co-implemented by researchers at the University of South Australia and key partners including a local council, a publicly funded primary school and an aged care service provider each based in Adelaide, South Australia. Despite the increasing popularity of intergenerational programs in recent years, there remains a limited understanding of the program specific components that lead to their success.

RE-AIM is an evaluation framework, often used in health promotion, and broad public health initiatives (19). Briefly RE-AIM incorporates five dimensions (Reach, Efficacy, Adoption, Implementation and Maintenance) that assess outcomes in relation to real-world translation and scaling of interventions (19, 20). Reach, is the proportion and characteristics of the target sample who received or were aware of the intervention. Efficacy, is any change (both positive and negative) in the main outcome measure or any secondary outcomes realized for the target population. Adoption, is the number and characteristics of the settings who adopted the planned intervention, with details on any barriers to participation also captured at the organizational level. Implementation, is the extent to which the intervention components have been implemented as originally planned, by adequately trained staff. Finally, Maintenance, assesses the ability to maintain and sustain the program over time (both individually and at the organizational/institution level).

Taken together, the predominant aim of this study was to evaluate the overall real-world sustainability and success of combining school-based dementia education with intergenerational excursions to guide future program development.

## Methods

### Participants

Three year 4/5 classes at a publicly funded co-educational primary school participated in an 8-week, school-based dementia educational program. Two classes were also invited to attend six intergenerational excursions held at a social-day program for older adults living in independently in the community with cognitive impairment (commonly dementia).

This study was approved by the University of South Australia Human Research Ethics committee (protocol no. 20070). All participants (children, older adults and staff) provided informed written consent. Where participants were unable to consent for themselves (e.g., children) they provided assent and consent was provided by a parent or legal guardian.

### Design

We employed a non-randomized, mixed methods, quasi-experimental evaluation approach whereby all students participated in dementia education lessons and two classes also attended the excursions. The allocation of classes to the excursion was non-randomized based on timetabling and excursion availability.

### Dementia Education Program

The dementia education program was modified and extended from the Kids4Dementia (K4D) content previously developed by teachers, children, people living with dementia, carers and academics (13). To complement K4D, other age-appropriate activities were developed by an education student designed around each main lesson theme (see, Table 1).

**Table 1:** Outline of the weekly topics discussed as part of the school-based dementia education program.

**Topic**	**Key resources needed**
Week 1: what is dementia?	• Assessment: KIDS Insight into Dementia Survey (KIDS) • Video: kids 4 dementia – module 1 (What is dementia?) • Activity booklet: introduction and title page
Week 2: communication and social interaction	• Video: Kids 4 dementia – module 6 (How does it feel to have dementia?) • Activity booklet: my letter plan
Week 3: environment	• Video: Kids 4 dementia – module 3 (What happens in nursing homes?) • Activity booklet: activity ideas mind map and activity poster
Week 4: memories	• Kids 4 dementia – module 4 (What causes dementia?) • Activity booklet: 3, 2, 1 response (3 dementia facts, 2 insights, 1 question)
Week 5: cognitive reserve	• Video: Kids 4 dementia – module 5 (How can we keep our brains healthy?) • Brain models to show children the different parts of the brain dementia can affect • Activity booklet: prevent dementia by poster proforma
Week 6: sensory changes	• Video: Alzheimer's society (Small changes make a dementia friendly world) and sony aibo technology example • Activity booklet: design technology to support people with dementia
Week 7: role of families and care staff	• Q + A session with a geriatrician • Brainstorm: how to make our environment dementia friendly?
Week 8: prevention (diet and lifestyle)	• Assessment: KIDS • Video: Kids 4 dementia – module 7 (How does it feel for the family?)

### Intergenerational Experience

The intergenerational experience was based around “the corner store” theme. This provided an opportunity for participants to discuss their experiences of shopping, and for the older adults, to share with the students how these shopping experiences had changed over their lifetime. Activities were led by Enabling Confidence at Home (ECH) activity and lifestyle staff, and commissioned community artists. Examples included flower pot planting, 8-ball, dyeing fabric (cyanotype) to make re-usable shopping bags, and group singing. Students were split into smaller groups to reduce noise throughout the center. Each student group were assigned a different activity each week, however, there was no structured grouping of children and adults.

### Program Rollout

Dementia education was led by University trained lecturers who had research expertise in dementia. One lesson was delivered each week (45 min) for 8 weeks in term 1 2018 (February – April).

Excursions began for two classes (*n* = 52 students) from week 3 of the program. Each excursion was 45 min in duration. The class that did not attend the intergenerational excursions (29 students) participated in similar art activities at the school, facilitated by ECH staff and community artists, but without the older adults.

### RE-AIM Measures

Table 2 presents the main outcomes for each RE-AIM dimension and participant group.

**Table 2:** RE-AIM components and associated outcome measures for the program.

**RE-AIM component**	**Outcome measure**
Reach	Individual Children: demographic questionnaire and baseline dementia knowledge assessment conducted in class during week 1 of program.
Efficacy	Individual Children: kids insight into dementia survey (KIDS). Overall score and factor scores for personhood, stigma and knowledge. Assessed immediately pre/post educational intervention. In addition, a subgroup of children attended semi-structured interviews with their parent/caregiver to discuss the broader impacts of the program. Parents: Semi-structured interviews with their child to discuss the family impact of the program, including any positive/negative changes in behavior they had identified in their child as a result of the program. Older adults: semi-structured interviews with older adults and their carers (where possible) to discuss their experiences of the program. Organizational Cost and benefit analysis conducted on the whole of program costs at the conclusion of program
Adoption	Organizational School: Number and percentage of classes offered the intervention who agreed to participate. Excursion location: Agreement of organizations approached to facilitate excursion.
Implementation	Organizational Assessed as the number of sessions (both education and excursion) that were delivered as intended. Measured by reports from key project staff.
Maintenance	Individual Children: Score on KIDS, 6-month post intervention (Term 4 2018). Organizational School & Aged care organization: intention and ability to continue with the program (either in full or component parts) after the completion of the intervention.

* *Individual and organizational refer to RE-AIM components assessed either individually or at the setting/organizational level*.

### Reach

Reach was assessed as the number of children who participated in the classroom lessons. Demographic characteristics (including previous dementia knowledge/familiarity) of the children were self-reported in class before and immediately after the program. Due to fluctuating attendance at the social day program it was not possible to assess the attendance of all older adults who attended the sessions. However, a core group were observed to participate during all sessions.

### Efficacy

To assess efficacy or change in children's dementia knowledge and attitudes the Kids Insight into Dementia Survey (KIDS) was completed individually under test conditions in a classroom environment at baseline, program completion and 6-month follow-up. KIDS provides good validity and internal consistency, strong concurrent validity and strong correlations with an adult measure of dementia attitudes in children aged 9–13 years (grades 4–7) (21). The 14 statements included in the KIDS are divided into three factors, personhood, stigma and knowledge. Six of the statements are negatively worded and were reverse-scored prior to analysis. Responses were summed to produce the total KIDS score, with higher scores indicating greater dementia knowledge and more positive attitudes (score range 14–70). Individual factor scores were also calculated for personhood, stigma, and knowledge. There was no true control group who did not receive dementia education. The school involved requested all three of their year 5 classes receive the educational content.

Following the removal of incomplete data and outliers, a one-way ANOVA was conducted with KIDS baseline total score as the dependent variable and knowledge or no knowledge of dementia as the independent variable (see Table 3). Subsequently, dementia knowledge or familiarity at baseline was included as a covariate in all models.

**Table 3:** Demographic characteristics of students participating in the dementia education program.

	**Excursion group (*n* = 37)**	**No excursion group (*n* = 22)**	**Overall (*n* = 59)**
Age (years)^*^	9.43 ± 0.56	9.95 ± 0.21	9.63 ± 0.52
Gender: male^∧^	65% (24)	50% (11)	59% (35)
Year 5 %^∧^	54% (20)	100.0% (22)	71% (42)
Lesson attendance %	97	94	96
Had dementia knowledge at baseline (amalgamated)^∧^	14% (5)	23% (5)	17% (10)
Heard about dementia^∧^	57% (21)	68% (15)	61% (36)
Seen someone with dementia^∧^	22% (8)	41% (9)	29% (17)
Watched movie/read book about dementia^∧^	27% (10)	36% (8)	30% (18)
Relative with dementia^∧^	11% (4)	9% (2)	10% (6)
Family friend with dementia^∧^	5% (2)	14% (3)	9% (5)
KIDS Baseline Score (Time 1) (range 14–70)^*^	50.0 (8.7)	52.55 (7.9)	51.0 (8.4)
KIDS post score (Time 2) (range 14–70)^*^	58.2 (7.1)	57.55 (6.5)	57.9 (6.8)
KIDS longitudinal score (Time 3) (range 14–70)^*^	59.1 (6.6)	59.3 (7.6)	59.2 (6.9)

* *m ± sd*,

∧ *% (n)*.

To investigate if the dementia education program was associated with a change in dementia knowledge and attitudes, the KIDS total and factor scores (for personhood, stigma and knowledge) were analyzed using separate mixed analyses of covariance (ANCOVA). The within-subjects factor was time (three levels: baseline, post-program and 6-month follow-up) and the between subjects factor was condition (two levels: excursion or no excursion). All significant main effects and interactions were explored with *post-hoc* pairwise comparisons adjusted for multiple comparisons with Bonferroni corrections. Normal distribution and homogeneity of variance of the data were assessed using the Kolmogorov-Smirnov test and Levene's statistic, respectively. Effect sizes were estimated with partial eta-squared (partial η^2^). In ANCOVAs where assumptions of sphericity were violated, the critical value of F was adjusted using the Greenhouse-Geisser epsilon value. Unless otherwise stated all analyses were performed in SPSS v25 (IBM, Microsoft Corporation). Significance was accepted at *p* < 0.05 (prior to any correction).

In addition to the primary outcome of the KIDS score, secondary outcomes for parents, older adults and carers were assessed through semi-structured qualitative interviews. Parents/guardians and children were interviewed at a neutral location (UniSA, City East Campus) during the school holidays immediately following program completion. Interviews typically lasted 20–30 min and covered topics such as expectations, impact, positives and negatives, potential improvements and examples of program translation to the home setting.

Older adults and their carers' were also interviewed at program completion either within their own home, over the phone or at ECH. Interviews included open-ended and probing questions to gain insights into reasons behind program participation, participant experiences (both positive and negative aspects were probed), suggestions for program improvement and noticeable changes in the older adults observed by carers or family members.

In addition to the interviews above, a cost benefit analysis of the program was also undertaken to provide a transparent method for assessing the “value for money” of the program. Cost data included: information on revenues received to run the program from Office of the Aging and additional in-kind resources reported from interviews with staff. Staff time was costed using data differentiated by Occupation from the Australian Bureau of Statistics Issue 6306.0 – Employee Earnings and Hours, Australia, May 2016. Prices were updated to 2018 prices using the Wage Price Index. Costs associated purely with the evaluation of the program were not included. The costs per unit of benefit were calculated by dividing the total cost of the program by the number of participants in the relevant groups.

### Adoption

Interviews with key staff from all contributing organizations were held at the completion of the intervention. Responses to questions pertaining to suggested improvements, successful components and barriers were used to assess adoption through thematic analysis.

### Implementation

Implementation was assessed by the project manager, who was onsite for all lessons and excursions. Lesson plans provided a reference for the lesson content that was delivered, and a checklist was developed for excursion activity stations. Key staff were also interviewed following the intervention to discuss issues with implementation at their respective organizations (as detailed in Adoption above).

### Maintenance

Individual level maintenance was determined as the 6-month follow up post scores on the KIDS survey for children. At the organizational level, semi-structured interviews with key staff identified the level of program related maintenance occurring at each organization as a result of the intervention.

## Results

### Reach

The three school classes had a combined enrolment of 87 students. Of these 81 parents provided consent for their child's data to be included in this evaluation (93% total reach). For equity purposes the remaining six children continued to participate in the program but their data were not used in the evaluation. Demographic characteristics for the children are presented in Table 3.

### Efficacy

#### Children

*Twenty* children reported having knowledge of dementia at baseline. Children who had knowledge or dementia familiarity at baseline (Table 3), performed better on the KIDS (*F*_[1, 76]_ = 8.38, *p* = 0.005).

**Figure 1:**
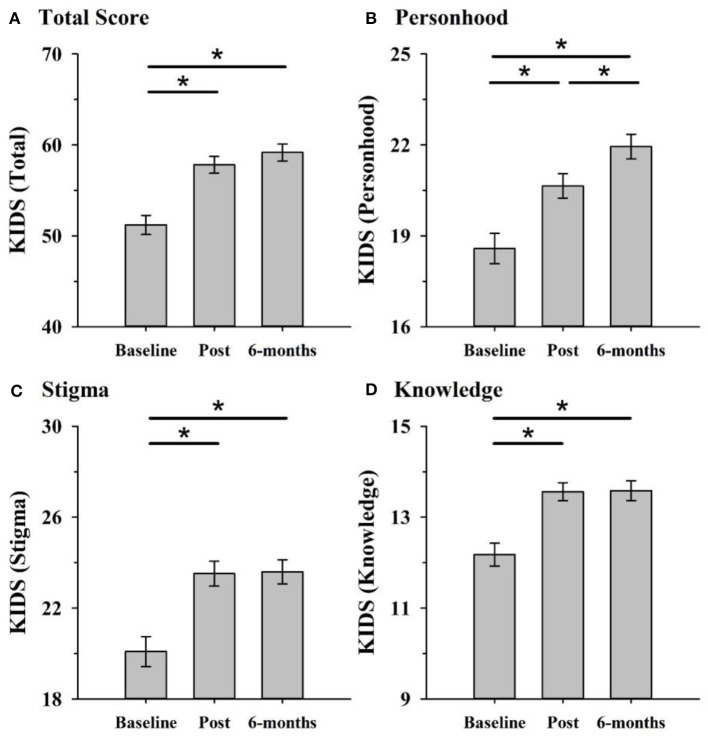
KIDS scores improved in both excursion and non-excursion classes after the program compared to before. Improvements were maintained at 6-months follow-up and occurred across the total score **(A)** and each of the three factors personhood **(B)**, stigma **(C)**, and knowledge **(D)**. *p < 0.05 (after Bonferroni correcting for multiple comparisons).

KIDS scores increased from baseline to post-program and was sustained at the 6-month follow-up (main effect of time: *F*_[1.8, 100.9]_ = 46.73, *p* < 0.001, partial η^2^ = 0.46). *Post-hoc* pairwise comparisons revealed improvement in dementia knowledge and attitudes from baseline to post-program (*p* = 0.001) and baseline to six-month follow-up (*p* < 0.001) (Figure 1) but no difference from post-program to 6-month follow-up (*p* = 0*.2*1). There was also a main effect of dementia knowledge at baseline (F_[1, 56]_= 5.79, *p* = 0.020, partial η^2^= 0.094) and a time x dementia knowledge interaction (F_[1.8, 100.9]_ = 3.24, *p* = 0.048, partial η^2^ = 0.06). There was no augmentation effect of condition (excursion or no excursion).

For each KIDS factor, there was a main effect of time: personhood (F_[2, 112]_= 28.32, *p* < 0.001, partial η^2^ = 0.34), stigma (F_[1.7, 95.86]_ = 29.07, *p* < 0.001, partial η^2^ = 0.34) and knowledge (F_[2, 108]_ = 22.08, *p* < 0.001, partial η^2^ = 0.29). For both stigma and knowledge, *post-hoc* pairwise comparisons revealed this was due to significant improvement from pre to post-program (both *p's* < 0.001), and pre to 6-month follow-up (both *p's* < 0.001), but not from post to six-months follow-up (both *p's* = 1.00). For personhood, scores increased from pre to post (*p* < 0.001) and long-term follow-up (*p* < 0.001) and also from post to 6-month follow-up (*p* = 0.007). There were no other main effects or interactions for the individual factors (Figure 1).

#### Parents

*Fourteen* parent and child dyads were interviewed directly after program completion to understand any wider secondary effects of the program on either the family unit, or directly on the child that were not assessed by the KIDS survey and to provide further information/benefits not identified in the KIDS survey. Following transcription and thematic analysis (22), Three of four identified themes pertinent to efficacy were positive changes and effects of completing the program, negative experiences These changes typically included children reporting being more patient with older relatives, and ability to explain the new knowledge gained to friends or family members, and greater empathy and patience in everyday situations. In some cases, children also identified their own improved behaviors (including increased patience and greater understanding while in public and with older family members) as a result of undertaking the program and in particular most children reported on the positive benefits of undertaking the excursions.

“*just being more careful around other people because they might have dementia but you can't tell by the way they look, or by the way they act.”* Child

“*I think that empathy even just hearing him say, just now, you know not taking people at face value and knowing that there might be underlying stuff.”* Parent.

#### Older Adults and Carers

*Twelve* older adults and four carers were also interviewed to discuss their experiences with the program. Overall, the intergenerational program was reported as positive, with many adults commenting on experiencing positive emotions, as a direct result of interacting with the children, and positive self-changes.

“*I really enjoy the children coming in. It's because I've had four of my own… and they say, you're a natural with kids”* Older Adult.

“*At Easter time, this beautiful boy made him [older adult] an Easter card. Oh, he was just about in tears when he came home”* Carer.

Whilst, there were few negative experiences reported, the large majority older adults reported high levels of noise during the excursions.

“*The only thing was the noise. That was about it. Otherwise no, there was no negative feedback at all”* Carer.

Some older adults and carers also reported an inability to remember activities that occurred during the program (this was particularly evident in the follow up period). This is likely to be due to a combination of interpersonal complications that occurred during the program, and the progressive nature of participants' condition, rather than a negative effect of the program.

“*You know sometimes I think I think I've been there, done that and it's out of your mind then, you know… But I can't recall that”* Older Adult.

#### Cost-Benefit Analysis

The costs associated with delivering the dementia education lessons and intergenerational excursions are estimated in Australian Dollars, AUD. These include both direct and in-direct costs and were estimated at $42,001 for the entire program. The direct costs incurred during the program included, set up and management costs ($2,919), school programming costs ($5, 000) and intergenerational program costs ($12,000). Direct program costs include estimations of implementing the art program (excursions), creating lesson plans and hire of charter buses. Indirect costs related to staff time used for program preparation ($17,064), and implementation of the program ($5,018). Costs for simply running the school-based dementia education program, without the intergenerational component were estimated at $7,919.

The cost-benefit analysis was conducted separately for the children who had data available post program (*n* = 70), and those who had data available at 6-month follow up (*n* = 59). Together per unit of student benefit was estimated at $600 per student who demonstrated an increase in dementia knowledge and attitudes at post program when compared to baseline (direct + indirect costs; Table 4). It is important to recognize that this estimate includes a significant proportion of in-kind support from staff in preparation and implementation of the program. This in-kind support equates to 52% of the total cost estimate with most of the in-kind support relating to preparation of the program. It is therefore likely that our initial cost benefit estimate of $600 per student who demonstrated an increase in dementia knowledge and attitudes represents an upper bound. This cost would also likely reduce, if more students had attended school on the day of the 6-month follow up testing. Unfortunately, due to public holidays and end of term, a number of students were absent on the day of 6-month testing and further testing days could not be rescheduled.

**Table 4:** Benefits and total costs per unit increase in dementia knowledge and attitudes in the children as a result of the program.

**Benefit**	**Number of participants who achieved the benefit**	**Direct costs for school program per unit of benefit**	**Direct costs (school program + excursions) per unit of benefit**	**Total cost per unit of benefit (direct + in kind)**
Students who participated across the three classes	88	$90	$226	$477 per student participating
Older people who participated	25	–	$796	$1,680 per older person participating
Students who demonstrated an improvement in dementia knowledge during the program	70	$113	$284	$600 per student with improved dementia knowledge
Students who demonstrated retention of improved knowledge and attitudes at long-term follow up	59	$134	$337	$711 per student with improved dementia knowledge

The cost-benefit analysis was also calculated examining only the school-related education component (excluding the excursions; Table 4). Costs per unit of benefit were estimated at $113 per student who demonstrated an increase in dementia knowledge and attitudes.

### Adoption

*One* school was approached to pilot this intervention and accepted on behalf of their year 5/6 cohort (three classes). The aged care partnership was more problematic, with two community based not for profit organizations offered the same opportunity. Both organizations provided initial quotes for their services (one for education component and one for art program) however due to the project requirements these partners withdrew their interest. A third partner was approached to assist with facilitating the excursions who accepted and was able to provide significant in kind staffing assistance to reduce the costs of excursions.

Interviews with key staff from all organizations were overwhelmingly positive. Staff recognized that the successful partnership between all four organizations (a council, school, aged care facility and university) was key to the success of the project as each partner provided their own lens in design, implementation and program support. Barriers that were raised throughout the interview series included noise at the center, the large number of children and subsequent high ratios of children to older adults (buddying or 2:1 preferable), and the distance to transport students to attend excursions.

### Implementation

Education sessions were provided separately for all three classes by trained university researchers. Educational content was mapped to curriculum priority areas and shared with teachers prior to lessons occurring (see Table 1). Sessions were supported as a timetabled fixture for the three participating classes with support from the school Principal. As such lessons were delivered as planned, despite any regular teacher absences (i.e., substitute teachers also supported the content delivery when required). Student workbooks used throughout lessons were also scanned for completeness to ensure educational content and activities were delivered as intended.

Similarly, excursions were also supported as a timetabled activity at the aged-care provider. With ECH agreeing to allow full access to their facility for the duration of the program, as well as sufficient time and staffing before and after sessions to allow for set up, and pack down of activity stations and art/music projects.

### Maintenance

Results from the follow-up KIDS survey indicated there were no decline in KIDS scores from post-program to 6-month follow-up (*p* = 0.21; Figure 1). This indicated that children's knowledge of dementia remained improved from the pre-program baseline, demonstrating a maintenance effect of the education. Organizational maintenance was assessed through key staff interviews at the conclusion of the program. Teaching staff indicated that they planned to continue with the theme of dementia education in later curriculum areas (in particular in relation to social studies activities). Staff at the aged-care provider were positive about the experience and would welcome the opportunity to be involved in similar projects in the future, however due to cost restrictions had no immediate plans to continue the intervention.

## Discussion

Overall this RE-AIM evaluation has provided strong evidence that dementia education improved children's knowledge and attitudes toward dementia, for at least 6 months. The combination of strong Reach, Adoption and Implementation resulted in significant positive changes in outcomes both immediately post-program, and in the 6-month follow-up. Interestingly, there were no between group differences in dementia knowledge or attitudes in the children who did or did not interact with older adults through the intergenerational experience.

Critical to the success of this program was the combined efforts and shared vision of all partner organizations who accepted invitations to participate. The ability to co-design both the lessons (with class teachers) and activity stations (with aged care staff) was one of the driving factors behind the successful implementation of the project. The co-location of settings, both in the school and in the aged-care facility also helped to ensure strong engagement from both the children and older adults, as they were familiar with the environment and viewed the intergenerational engagement as novel and exciting.

These outcomes align strongly with the aims of the World Health Assembly's Global Action Plan on the public response to dementia 2017–2025 (23). In particular, increasing public dementia awareness, and establishing a dementia friendly society through public awareness campaigns have been identified as important future actions. Our findings here support the embedding of dementia education into the school curriculum as one strategy that facilitates long-term improved knowledge and reduces stigmatization of people living with dementia in a segment of the population who will be future leaders, business owners and health care workers.

The strong maintenance effect at the individual level, as seen in this program is another key strength. It is possible that the detailed program of education, where the children received 6 h of education across 8 weeks led to this effect (45 min × 8 lessons). Across the 8 weeks, children were exposed to a range of topics including communication, environment, memories, cognitive reserve, sensory changes and prevention (Table 1). In contrast, dementia education/training in healthcare settings is not mandatory or consistently delivered (24). A recent systematic review identified only 14 studies investigating dementia education for health professionals within general hospital settings. Each of the included studies varied in terms of the program development and delivery, and none included a long-term follow-up (24). Programs varied in length from 2 h of education (25) to 12 days (26). Likewise, dementia education is also delivered inconsistently for pre-registration health care trainees (27). In their review (27), concluded dementia education programs were not consistently undertaken for health care trainees and most were conducted with undergraduate nursing students, whereas only three programs were conducted with medical trainees. Importantly, the most effective programs did not rely on theoretical input alone, but included both theoretical learning, and practice-based experience, by encouraging interactions between students and people living with dementia. The programs employing this combined approach resulted in increased student comfort to interact with people living with dementia, and improvements in confidence and communication at post program compared to pre. Taken together, with our qualitative findings, this body of evidence suggests that whilst improvements in knowledge and attitudes toward dementia can occur with education alone, practical interactions with people living with dementia are critical to increase confidence and enhance communication skills.

It was interesting to note, intergenerational experiences alongside the dementia education lessons did not have cumulative benefits for children's knowledge and attitudes. This suggests that dementia education alone is enough to change children's knowledge and attitudes in the following 6-months. Qualitative interviews with parents and children provided further important insights into the role of the intergenerational experience that were not captured with the KIDS. For example, interviewed parents reported additional benefits directly attributed to the intergenerational excursions such as: improvements in empathy, and reductions in children's negative judgement of older people and people living with dementia, within the community. Assessment of these additional benefits were not specifically targeted in the KIDS. One key difficultly commonly reported in intergenerational literature is how best to operationalise the benefits attributed to intergenerational interactions (12, 28).

The cost burden of these types of studies was the main barrier for future implementation raised by the non-adopters of our program. While our calculations show a $600 AUD cost per student who demonstrated an increase in dementia knowledge/attitudes, there are alternative approaches to assist in reaching an economy of scale. For instance, program preparation has now occurred, with educational content created and mapped to the curriculum. Variable costs including student transport could be reduced or eliminated if school and providers were in close proximity to each other. It is important to note that some fixed costs will remain to ensure participant safety, such as staff to student/older adult ratios.

The costs incurred per child appear to be within the range of similar programs run in pre-school or primary schools in Australia. Total costs for the Cool Little Kids intervention (designed to prevent anxiety and depression in preschool age children and incorporating six 90-min face to face group sessions with a psychologist) were similar at $549 per child (29). The intervention led to a reduction in the number of children diagnosed with anxiety (44.2 vs. 50.2%). Total costs for a school-based healthy eating and physical activity education program in high school students were $1,388 per student (30). Benefits included an increase of 5.2% of students eating more than 2 serves of fruit per day, and a 2.5% increase in students eating more than 4 serves of vegetables per day.

By comparison, the costs appear higher than a volunteer-driven multicomponent intervention for people with dementia and their caregivers (£75 per dyad) (31). However, there is evidence that the costs of programs for people with dementia can reduce overtime from the start-up phase to the continuing phase of the program (likely due to economies of scale and increased efficiency once the program is up and running). For example, the costs of care-coordination programs in people with dementia decreased from between $501–$581 during the start-up phase for the program, to $142–$241 per participant per month once the programs were up and running in a stable phase (all in US dollars) (32). Therefore, similar economies of scale and increased efficiency may occur if the dementia-education program were run again in multiple regions.

Key strengths of our program included its co-design with council, aged care and primary school teachers, co-implementation and evaluation based on the RE-AIM framework. A potential limitation not considered or controlled for in the current study, was how the classroom teachers extended and applied extra dementia education across other aspects of the curriculum, outside of the dementia lessons each week. Indeed, it is possible that the class not attending the excursions were exposed to more dementia education delivered by their classroom teacher, outside of the weekly lessons so the students did not feel disadvantaged by missing out on excursions each week. This may potentially account for the lack of differences we observed between the excursion and non-excursion classes. In future, controlling for, or keeping a record of how much time teachers spent discussing dementia, outside of the program should be considered. Future studies could also consider implementing the program across multiple schools instead of having the excursion and non-excursion groups within the same school.

### Implications for Future Research

Whilst we show here that dementia education is sufficient to improve children's knowledge and attitudes 6-months after the completion of the program. It is possible that the specific benefits of the intergenerational excursions were not fully captured in our chosen primary outcome assessment. Building on the novel findings, future studies should consider further refining the intergenerational formats based on identified successful elements of other intergenerational dementia programs (28, 33–35). Taking the findings of our study with the existing extant literature (36, 37), four key successful elements could be considered for future intergenerational programs including (1) buddy systems to foster relationship building; (2) embedding dementia education within intergenerational experiences; (3) considerations around activity set-up (based on participant abilities and preferences); and (4) analysis of student reflective journals to gain a greater insight into the holistic program benefits. It is noted that the sample size of the study is relatively small, and the program conducted only within one primary school and age care facility. An obvious future avenue for research would be to investigate how best to upscale school-based dementia education more broadly including modifying the program for different age-groups. This was beyond the scope of the current study.

Using a RE-AIM evaluation approach, our findings suggest that 8-weeks of school-based dementia education can be successfully presented and implemented in a community setting. Such a program can significantly improve children's knowledge and attitudes toward dementia for at least 6 months. Importantly, improvements were seen for the total KIDS score as well as each of the individual factor scores of personhood, stigma and knowledge and no differences in knowledge or attitudes toward dementia occurred between the groups of students who interacted with older adults and those who did not. Given the absolute increase in the number of people living with dementia worldwide, programs of this nature will be important to improve dementia knowledge and reduce dementia-related misinformation and stigmatization of people living with dementia.

## Data Availability Statement

De-identified datasets generated for this study are available on reasonable request to the corresponding author.

## Ethics Statement

The studies involving human participants were reviewed and approved by University of South Australia Human Research Ethics Committee. Written informed consent to participate in the study was provided by all participants. Where participants could not consent for themselves (e.g., children) written assent was obtained from the children and consent to participate in the study was provided by the participants' legal guardian/next of kin.

## Author Contributions

AS planned the study, supervised the data analysis, and drafted the manuscript. GK, SL, and MH undertook data collection, cleaned the data, performed all statistical analyses, and contributed to revising the paper. JB helped plan the study, informed the educational program, and revised the manuscript. RM and JR supervised the economic analysis and revised the manuscript. TL and HK helped plan the study, contributed to educational program delivery, and revised the manuscript. All authors contributed to the article and approved the submitted version.

## Conflict of Interest

The authors declare that the research was conducted in the absence of any commercial or financial relationships that could be construed as a potential conflict of interest.
